# Undiagnosed diabetes mellitus and associated factors among adults in Ethiopia: a systematic review and meta-analysis

**DOI:** 10.1038/s41598-021-03669-y

**Published:** 2021-12-20

**Authors:** Getachew Yideg Yitbarek, Gashaw Walle Ayehu, Sintayehu Asnakew, Ermias Sisay Chanie, Wubet Alebachew Bayih, Dejen Getaneh Feleke, Tadeg Jemere Amare, Fentaw Teshome, Assefa Agegnehu Teshome, Getachew Arage, Fanos Yeshanew Ayele, Alemayehu Digssie Gebremariam, Melaku Tadege Engidaw, Sofonyas Abebaw Tiruneh

**Affiliations:** 1 Department of Biomedical Sciences, College of Health Sciences, Debre Tabor University, Debre Tabor, Ethiopia; 2 Department of Psychiatry, College of Health Sciences, Debre Tabor University, Debre Tabor, Ethiopia; 3 Department of Pediatrics and Child Health Nursing, College of Health Sciences, Debre Tabor University, Debre Tabor, Ethiopia; 4 Department of Public Health, College of Health Sciences, Debre Tabor University, Debre Tabor, Ethiopia; 5 Department of Public Health, College of Health Sciences, Wollo University, Dessie, Ethiopia

**Keywords:** Physiology, Endocrinology, Health care

## Abstract

Diabetes has become a major public health problem, with 4.6 million deaths annually. The number of people living with undiagnosed diabetes is on the rise and has a diverse prevalence. Thus, this systematic review and meta-analysis was aimed to synthesize the pooled estimate prevalence of undiagnosed diabetes mellitus, impaired fasting glucose and its associated factors in Ethiopia. The databases Medline, Hinari, Google Scholar, and Google search were used to find potential studies published from January 2013 until January 2021. Extracted data were entered into the excel spreadsheet. The random effects model with Der Simonian-Laird weights was used to assess the pooled estimate of prevalence of undiagnosed diabetes, impaired fasting glucose, and its associated factors. The Cochrane Q-test and I^2^ statistics were used to screen for statistical heterogeneity. A funnel plot and Egger's statistical test were also used to search for any publication bias (small study effect). After extensive searching of articles on different databases, a total of nine studies were included for this systematic review and meta-analysis. In random effects model, the pooled prevalence of undiagnosed diabetes mellitus and impaired fasting glucose was 5.75%, 95% CI (3.90–7.59%), and 8.94%, 95% CI (2.60–15.28%), respectively. Regarding the associated factors, participants family history of diabetes was significantly associated with diabetes status. The pooled odds of developing diabetes mellitus among participants with a family history of diabetes mellitus were about 3.56 times higher than those without a family history of diabetes mellitus (OR = 3.56, 95% CI (2.23, 5.68)). In this review, the higher prevalence of undiagnosed diabetes mellitus and impaired fasting glucose was observed among adults in Ethiopia. Family history of diabetes was found to have an association with increased risk of diabetes mellitus. Our finding highlights the need of screening at the community level, with special focus on adults with family history of diabetes mellitus.

## Introduction

Diabetes mellitus (DM) is a metabolic condition caused by irregularities in insulin secretion, insulin function, or both, which causes chronic hyperglycemia^1^. It is a broad term for a group of diseases caused by a variety of causes^2^. The term pre-diabetes is a form of metabolic syndrome marked by elevated blood sugar levels that are below the diabetes mellitus diagnostic threshold^1^. Hemoglobin A1C, fasting glucose, or a glucose tolerance test can all be used to diagnose pre-diabetes^2^. Impaired fasting glucose (IFG) is a disorder in which fasting blood glucose levels are higher than what is considered normal^3^.

According to world health organization (WHO) global status report, diabetes mellitus is one of the top ten causes of disease in the world^4^. If nothing is intervening, the number of diabetics is expected to increase from 366 million in 2011 to 552 million by 2030. Diabetes is linked to a number of chronic noncommunicable illnesses (NCDs), including neuropathy, heart disease, kidney disease, and vision impairment, as well as depression and amputation^5^. Costs are projected to rise as the diabetes pandemic spreads. The medical costs of managing diabetes-related complications and comorbidities are one cause for such high diabetes-related spending. Diabetes complications have also been shown to reduce the quality of life of diabetic patients and increase their risk of disability or death^6^. Moreover, it has a negative impact on productivity and human growth^7^. Evidences revealed that many adults in developing countries including Ethiopia are suffering from the disease without ever being diagnosed^8^. The economic burden of diabetes has an impact on the health-care system, as well as on the affected individuals and their families. DM imposes a catastrophic out-of-pocket personal expenditure from loss of income owing to disability and premature death, in addition to the fiscal load on the health-care system and the national economy^9^. Population-based survey carried out in China, revealed that prevalence of DM was 4.19% and proved that early detection and proactive treatment are undeniably important to prevent new cases from emerging^10^. Previous studies in Turkey and Nigeria showed that prevalence of undiagnosed diabetes was 1.1 % and 0.8% respectively^11,12^.

The estimated prevalence of DM in adult population of Ethiopia is 1.9%^13^. Another study conducted in north Ethiopia showed that the prevalence of IFG were 12%^14^. Previous Studies conducted in different areas showed that age, family history of diabetes, hypertension, overweight, low physical exercise activity and alcohol consumption were considered as risk factors for the development of diabetes mellitus^15–17^.

To the best of our knowledge, there is a scarcity of systematically synthesized data on the prevalence of undiagnosed diabetes mellitus and its associated factors, especially in Ethiopia. he implications behind this review include providing public health professionals with detailed knowledge to deliver health promotion and disease prevention strategies, as well as enabling the early identification of adults at risk of diabetes mellitus in the future. It will also provide researchers with knowledge to help them identify research gaps and direct future research progress. Thus, The aim of this systematic review and meta-analysis was to provide the most up-to-date scientific evidence on the pooled prevalence of undiagnosed diabetes mellitus, IFG, and its related factors in Ethiopia.

## Methods and materials

### Study setting and searching strategies

All methods were performed in accordance with the relevant guidelines and regulations. We conducted a literature search on Medline, Hinari, Google Scholar, and Google databases for studies published from January 2013 up to January 2021. In addition to looking for unpublished theses, certain research centers and libraries sources have been searched out. All searches were restricted to English-language studies. Medical subject heading (MeSH) terms ((((("Magnitude") OR "prevalence") AND "Undiagnosed diabetes mellitus") OR "impaired fasting glucose") AND "Associated factors") AND Ethiopia AND Human) were used in various combinations as primary search keywords.

### Eligibility criteria

For prevalence studies, we used the CoCoPop (Condition, Context, and Population) method to announce inclusion and exclusion criteria.

### Inclusion criteria and exclusion criteria

All studies conducted in Ethiopia on the prevalence and factors linked to undiagnosed diabetes mellitus and impaired fasting glucose were included. Furthermore, all full-text English-language publications with research conducted among adults aged 18 and above, as well as studies published after January 1, 2013, were qualified to be included in this systematic review and meta-analysis. Studies with no prevalence study on undiagnosed diabetes mellitus, studies with insufficient data on desired information, and studies conducted on known DM patients were omitted from this analysis. We contacted authors when necessary and manually checked all listed publications' reference lists as well as recent systematic reviews. On the topic, books, chapters, and review articles were also consulted.

### Measurement of the outcome variables

The primary outcome of interest for this review was to estimate the pooled prevalence of undiagnosed DM and its associated factors in Ethiopia. Undiagnosed DM was defined as an individual whose diabetes has not been diagnosed by a physician but whose plasma glucose levels satisfy established criteria for diabetes, for instance a fasting blood glucose level of greater than or equal to 126 mg/dl as per WHO classification.

### Study selection and data collection

Endnote version X9.2 (Thomson Reuters, Philadelphia, PA, USA) program was used to combine, export, and handle all of the studies found across various databases. All duplicate studies were deleted, and the full text of the papers was manually and electronically searched using Endnote software. Individual article eligibility was determined independently by the reviewers (GY, GW, MT, DG, WA, ES, SA & AA) based on a review of the title, abstract, and full text. Discussion and consultation with a third reviewer resolved the difference between reviewers.

### Data extraction

The following information was gathered and documented on a standardized form that was used to document related items and entered into a database (Microsoft Excel): the proportion of participants with undiagnosed DM and IFG with standard error, research characteristics (authors, year of publication, region, study design, environment, mean age of participants, gender) were reported. Another reviewer double-checked the accuracy of the extracted data.

### Quality assessment of individual studies

The quality of included studies were assessed using the modified version of a quality assessment tool for prevalence studies which is validated in the previous study. Reviewers (SA, FT, FY, AT, AD, TJ, GA & WA) independently assess the quality of the included studies. The discrepancy between the reviewers were managed trough discussion and articles were included after consensus. The quality assessment tool measures a total of nine questions. Based on the score of the quality assessment tool, the highest score from nine questions declared low risk of bias. Overall scores 0–3, 4–6, and 7–9 were declared low, moderate, and high risk of bias respectively^18^ (Table 1).

**Table 1:** Quality assessment of individual studies included in systematic review and meta-analysis on prevalence and associated factors of Undiagnosed DM in Ethiopia, 2021.

Authors	1	2	3	4	5	6	7	8	9	10	11
	Target group representative	Study pop representative	Census or random	Low non-response bias	Primary data collection	Acceptable case definition	Appropriate instrument measurement	same model of data collection was used	Proper calculation of prevalence	Total 'yes'	Overall risk of bias
Bantie et al.	Yes	Yes	Yes	Yes	Yes	Yes	Yes	Yes	Yes	9	0
Abebe et al.	Yes	Yes	Yes	Yes	Yes	Yes	Yes	Yes	yes	9	0
Ayele et al.	Yes	No	Yes	Yes	Yes	Yes	Yes	Yes	yes	8	1
Endris et al.	Yes	Yes	Yes	Yes	Yes	Yes	Yes	Yes	yes	9	0
Wolde et al.	Yes	Yes	Yes	Yes	Yes	Yes	Yes	Yes	no	8	1
Worede et al.	Yes	Yes	Yes	Yes	Yes	Yes	Yes	Yes	yes	9	0
Dereje et al.	No	Yes	Yes	Yes	Yes	Yes	Yes	Yes	yes	8	1
Tesfaye et al.	No	Yes	Yes	Yes	Yes	Yes	Yes	Yes	Yes	8	1
Wondimagegn et al.	Yes	Yes	Yes	Yes	Yes	Yes	Yes	Yes	Yes	9	0

### Statistical analysis

A format prepared in Microsoft Excel spreadsheet was used to extract the appropriate details from each original report. For further review, the extracted data was exported to STATA/MP version 16.0 software. The random effects model with Der Simonian-Laird weights was used to assess the pooled estimate of prevalence of undiagnosed DM, IFG, and its related factors^19^. The Cochrane Q-test and I^2^ statistics were used to screen for statistical heterogeneity^20^. Subgroup analysis based on the mean age of the participants was used to reduce the variance of point estimates between primary studies. A sensitivity analysis was also performed to see how single studies affected the pooled estimate. A funnel plot and Egger's statistical test were used to search for publication bias (small study effect). The existence of a small study effect is treated by non-parametric trim and fill analysis using the random effects model when the p-value is less than 0.05^21^. The pooled effect was expressed as an odds ratio to classify variables linked to the outcome variable.

## Results

### Characteristics of the studies

**Figure 1:**
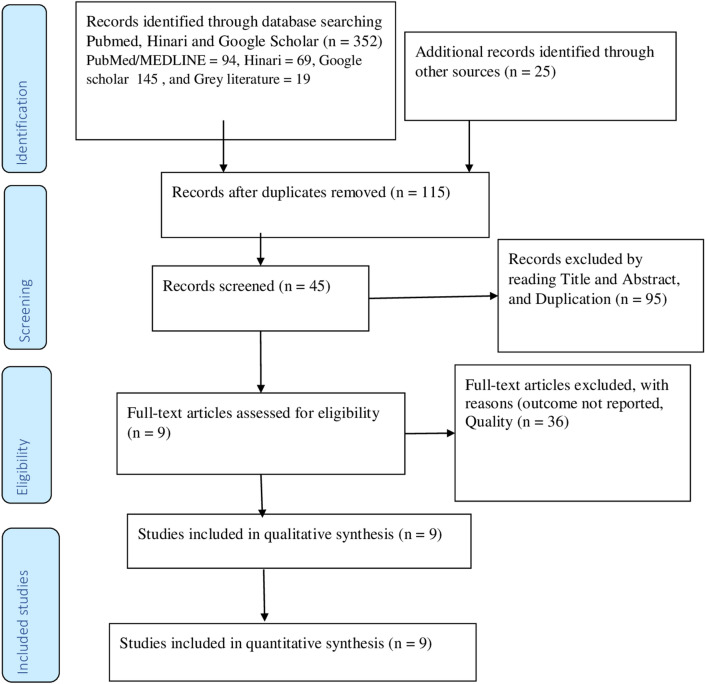
PRISMA flow diagram of article selection for systematic review and meta-analysis of magnitude of undiagnosed DM and associated factors in Ethiopia, 2021.

A total of 352 articles were identified in the literatures search. After removing duplicates 115 articles were left. Further screening by title and abstract against the review objectives and inclusion criteria, 95 articles had been excluded. The full texts of the remaining 36 articles were again excluded. Finally, 9 studies were included in the current systematic review and meta-analysis. The flow diagram of the study search and selection process are depicted using Preferred reporting items for systematic reviews and meta-analyses (PRISMA)^22^ (Fig. 1).

Many of the studies included in this review were conducted after 2013 and are cross-sectional in nature. The overall sample size for the studies was 7664, with all participants being over the age of 18. The published sample sizes for the minimum and maximum sample sizes were 392 and 2141, respectively^14,23^. Undiagnosed DM prevalence was registered as low as 2.05% and as high as 11.5% in the SNNP and Amhara regions, respectively^24,25^. For this systematic review and meta-analysis, potential studies were retrieved from only four regions. Six from Amhara^8,14,23,25–27^, one from SNNP^24^, one from Dire Dawa^28^ and one from Addis Ababa^29^ were included (Table 2).

**Table 2:** Characteristics of the studies included for systematic review and meta-analysis on prevalence and associated factors of Undiagnosed DM in Ethiopia, 2021.

S. no	Author	Publication year	Region	Sample size	Diabetes assessment Criteria	Prevalence of undiagnosed DM	Quality score
1	Endris et al.	2021	Amhara	587	WHO	4.9	7
2	Dereje et al.	2020	SNNP	634	WHO	2.05	6.5
3	Wolde et al.	2020	Amhara	773	WHO	5.1	6.5
4	Ayele et al.	2020	Dire Dawa	872	WHO	6.2	7
5	Bantie et al.	2018	Amhara	607	WHO	10.2	7
6	Worede et al.	2017	Amhara	392	WHO	2.3	7
7	Tesfaye et al.	2016	Addis Ababa	936	WHO	3.41	6.5
8	Wondimagegn et al.	2016	Amhara	722	WHO	11.5	6.5
9	Abebe et al.	2014	Amhara	2141	WHO	7.2	7

### Prevalence of undiagnosed diabetes mellitus in Ethiopia

**Figure 2:**
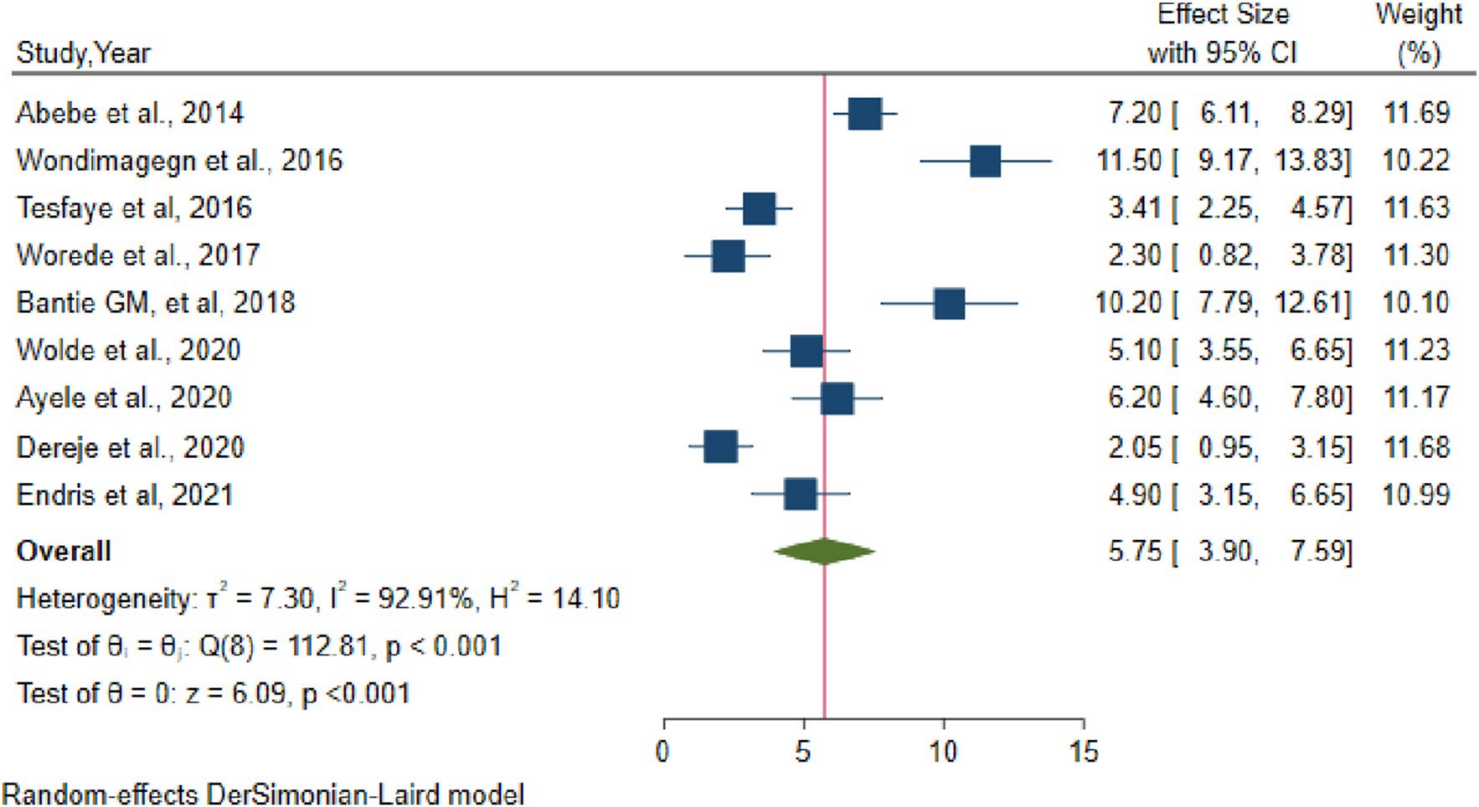
Forest plot of pooled prevalence of undiagnosed diabetes mellitus in Ethiopia, 2021.

**Figure 3:**
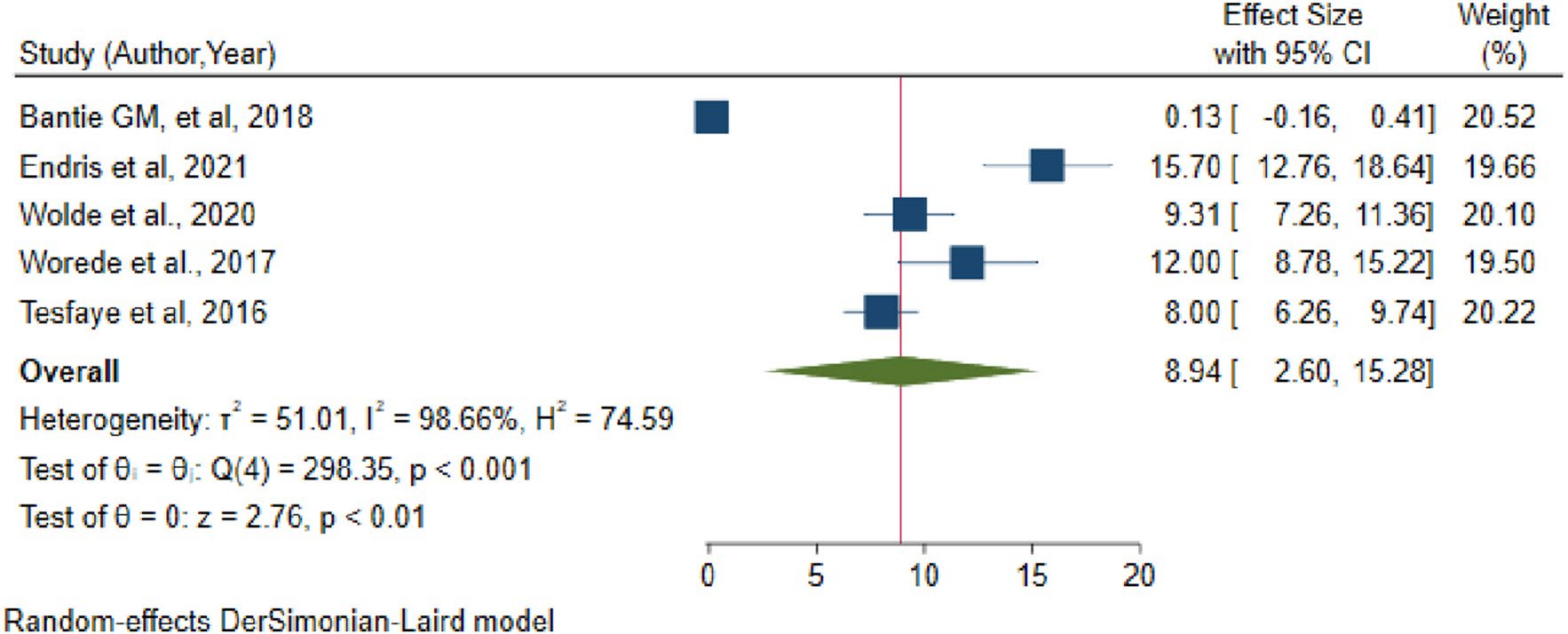
Forest plot of pooled prevalence of impaired fasting glucose in Ethiopia, 2021.

To estimate the pooled prevalence of undiagnosed diabetes mellitus in Ethiopia, nine studies were included. In random effects model, the pooled prevalence of undiagnosed diabetes mellitus in Ethiopia was 5.75 (95% CI 3.9–7.59). Significant heterogeneity was observed among studies (I^2^ = 92.91, p-value < 0.001). The highest weight among studies was observed from studies conducted by Dereje et al., and Abebe et al., (Fig. 2). Egger's statistical test was used to analyze a small study effect (publication bias). The included studies passed Egger's statistical test with a p-value of 0.165, indicating that there was no publication bias. On the otherhand, five studies have been included to estimate the pooled prevalence of IFG in Ethiopia. In random effects model, the pooled prevalence of impaired fasting glucose in Ethiopia was 8.94 (95% CI 2.6–15.28) (Fig. 3). Although there was significant heterogeneity among studies (I^2^ = 98.66, p-value 0.001), sensitivity analysis revealed that no single study influenced the pooled estimated prevalence of IFG in Ethiopia.

### Handling heterogeneity

**Figure 4:**
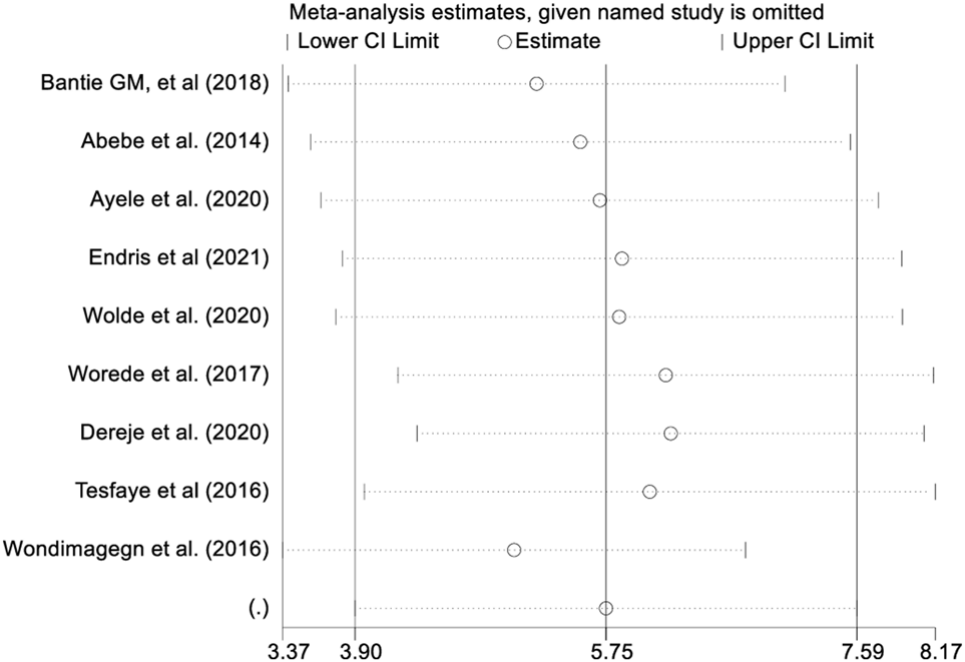
Sensitivity analysis of the studies included in systematic review and meta analysis on prevalence of undiagnosed diabetes mellitus in Ethiopia, 2021.

**Figure 5:**
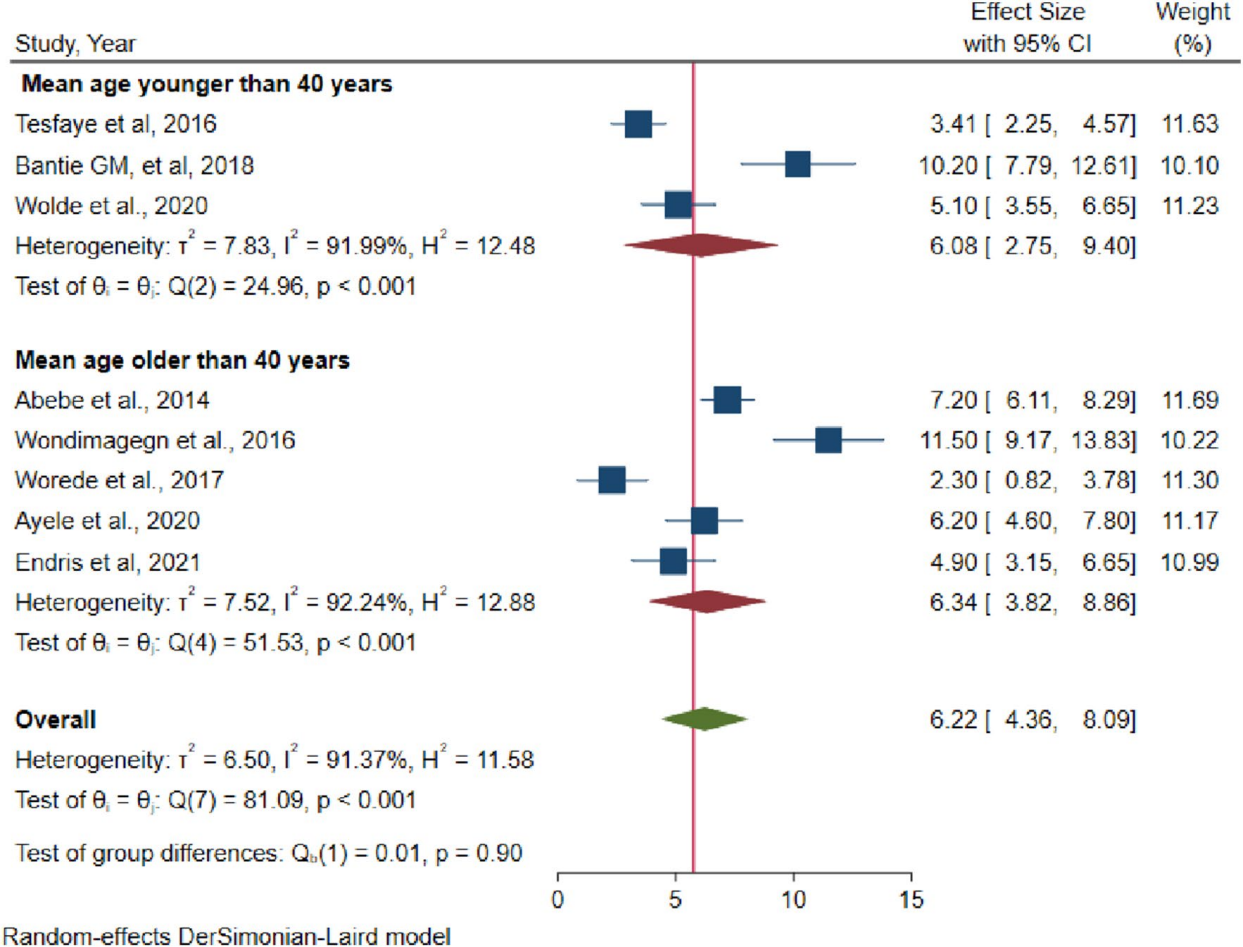
Subgroup analysis of the studies included in systematic review and meta-analysis on prevalence of undiagnosed DM in Ethiopia, 2021.

The pooled estimate from the random effects model revealed substantial heterogeneity. For the pooled estimation of undiagnosed DM, sensitivity analysis and subgroup analysis were performed to account for this heterogeneity. From the random effects model, there are no studies excessively influenced the overall pooled estimate of prevalence of undiagnosed DM among the included studies (Fig. 4). Despite subgroup analysis was performed based on the participants' mean age, the heterogeneity between studies did not change. The pooled estimate prevalence of undiagnosed DM was 6.08 (95% CI 2.75, 9.4) among study groups younger than 40 years old, whereas it was 6.34 (95% CI 3.82, 8.86) among study groups older than 40 years old, according to the subgroup review (Fig. 5). In the subgroup analysis, the highest prevalence of undiagnosed DM were observed in Abebe et al. followed by Tesfaye et al. (Table 3).

**Table 3:** Subgroup pooled prevalence of undiagnosed DM based on age of the participant in Ethiopia, 2021 (n = 9).

Variables		Included studies	Sample size	Prevalence (95%CI)	Heterogeneity (I^2^, p-value)
By mean age	≤ 40	4	2316	6.08 (95%CI 2.75, 9.4)	91.99%, p < 0.001
	> 40	5	5348	6.34 (95%CI 3.82, 8.86)	92.24%, p < 0.001

### Factors associated with undiagnosed diabetes mellitus

A separate random effects model pooled estimate analysis was employed on the extracted factors (family history of DM, age, sex and BMI) to identify the determinant factors of undiagnosed DM in Ethiopia. However, only family history of DM was found significant associated with the development of DM (Table 4*)*.

**Table 4:** Summary of the pooled effects of factors associated with undiagnosed DM among adults in Ethiopia, 2021.

Variables		OR (95% CI)	Heterogeneity (I^2^, p-value)	Egger’s p-value	Number of studies	Sample size
Family Hx of DM	No	1				
	Yes	3.56(2.23, 5.68)*	48.94%, 0.08	0.7	6	5571
BMI	18.5–24.5	1	1			
	< 18.5	1.34 (0.97, 1.84)*	0.85%, 0.35	0.6	6	5571
	> 24.5	1.36 (0.72, 2.1)	26.4%, 0.25	0.9		
Sex	Male	1	1			
	Female	1.81 (0.71, 4.01)	70.2%, < 0.001	0.56	9	7664
Age	Below 40	1	1			
	≥ 40	3.6 (0.99, 7.8)	87%, < 0.001	0.8	9	7664

**Figure 6:**
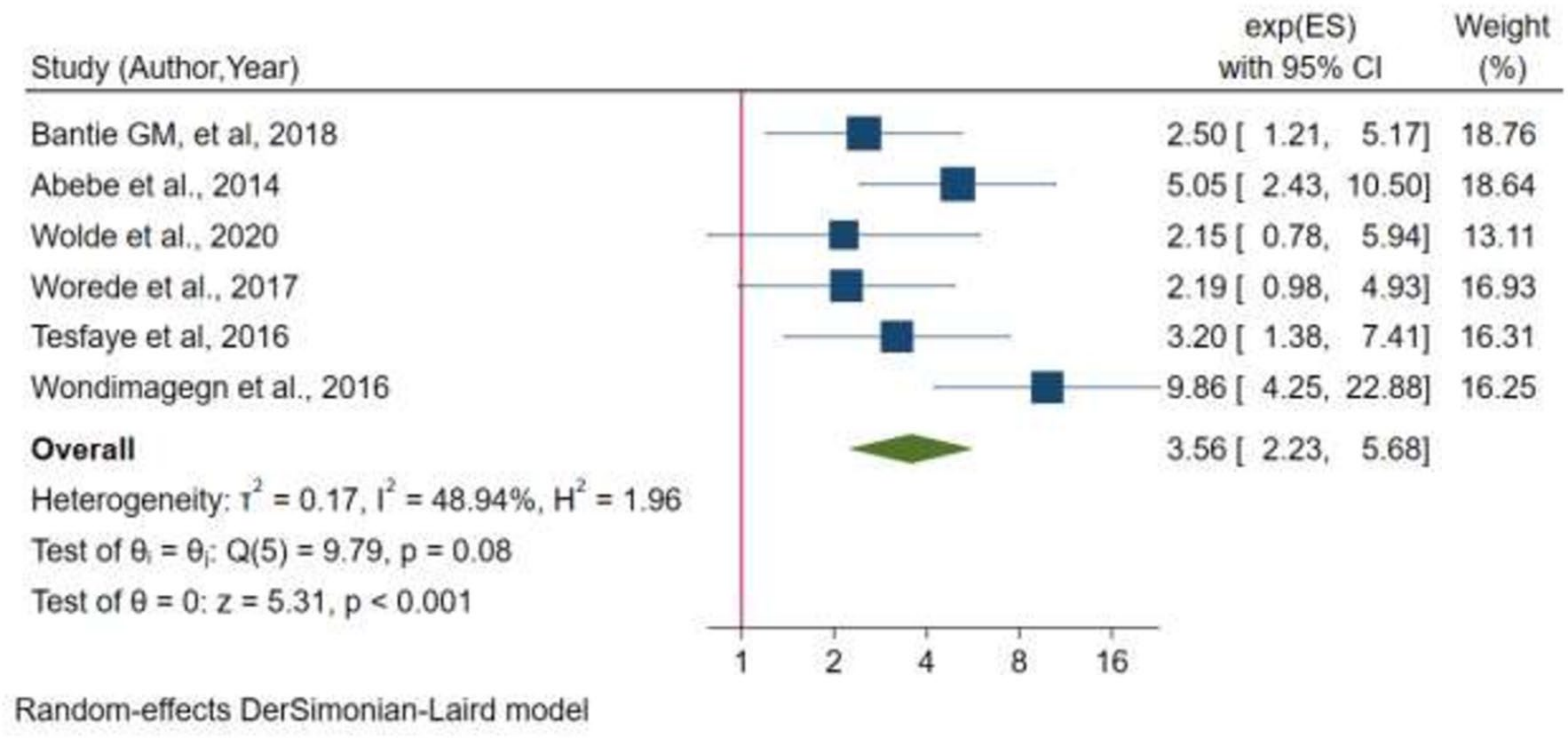
Forest plot for the association between family history of DM and diabetes mellitus, a systematic review and meta analysis in Ethiopia, 2021.

Six studies were included in the analysis to see whether there was an association between participants' diabetes status and their family history of diabetes. Two of the six studies found no association between diabetes and participants family history. The pooled odds of developing diabetes mellitus among participants with a family history of DM were 3 times higher than those without a family history of DM, according to random effects model estimates (OR = 3.56, 95% CI 2.23, 5.68) (Fig. 6). There was no statistically significant heterogeneity between studies (I^2^ = 48.94%, p-value = 0.08) There is no small study effect (p-value = p = 0.29) and no single study that affects the pooled estimate from sensitivity analysis, according to Egger's statistical test calculation.

## Discussion

There is also overwhelming evidence that the prevalence of diabetes is rapidly increasing in many developing countries in which undiagnosed diabetes is more likely to be associated with severe diabetic complications including diabetic neuropathy, cardiovascular disease and diabetic retinopathy^30,31^. Therefore, this systematic review and meta-analysis aimed to give updated pooled prevalence of undiagnosed DM and IFG in Ethiopia.

This systematic review and meta-analysis revealed that the pooled prevalence of undiagnosed DM in Ethiopia was 5.75, 95% CI (3.9–7.59). The pooled estimate of DM was in line with previous studies conducted in Canada 5.6%^32^, pooled estimate of African population 5.37%^33^, Teheran, Iran 4%^34^ and Qatar 5.9%^35^. The prevalence detected in this study (5.75%) was also greater than Ethiopia's projected countrywide prevalence (3.32%) published by the IDF in 2012^36^. This may be associated with the global increase in the trend of DM and the predicated epidemic in developing countries.

However, the finding of this meta-analysis was lower than a previous studies in East Gojjam zone, (11.5%)^25^,Germany (8.2%)^37^, Malaysia (8.9%)^38^, and Kenya (14%)^39^.The difference could be attributable to the sociodemographic and lifestyle variation between the study areas. In addition, the measurement tool used to determine fasting blood glucose level by the other studies was HbA1c, which was more reliable, whereas in our case Ethiopia, it was based on a single measurement, which might fluctuate due to different factors.

On the other hand, the pooled prevalence of Undiagnosed DM in Ethiopia was higher than previous study conducted in Koladiba (2.3%)^14^, Sothern Ethiopia (2.05)^24^, Nigeria 0.8%^40^, and Congo 1.5%^41^. This could partly be due to differences in the composition of study population. Additionally, the inconsistency would be related with the sample size used to conduct the study.

This systematic review and meta-analysis also identified that the pooled estimate of magnitude of IFG was 8.94, 95% CI (2.6,15.28), showing that unless appropriate interventions were made, the individuals might develop diabetes. The finding was consistent with population based study in Qatar (12.5%)^35^, Bahirdar (12.8%)^27^. In contrary to this, the pooled estimate of IFG in Ethiopia is lower than previous study in Uganda (20%)^42^, Indonesia (58.8%)^43^, and Brazil (18.4%)^44^ while it is higher than previous study in Qatar (1.3%).This could be due to the age difference and the study setting where in Ethiopia only urban residents are included in the study.

The subgroup analysis showed that the pooled estimate prevalence of undiagnosed DM was higher among group of studies which with mean age greater than 40 years of age which is 6.34 (95% CI 3.82, 8.86). This is due to the fact that as age increase the likelihood of developing chronic illness is higher.

This systematic review and meta-analysis also identified the determinant factors of undiagnosed DM in Ethiopia. In random effects model pooled estimates, family history of diabetes mellitus (FHDM) was significantly associated with the development of DM. From the random effects model estimate, the pooled odds of developing DM among participants with family history of DM were nearly 3.5 times higher than those participants without FHDM. This is supported with many studies conducted in different parts of the world including China^30^, Uganda^42^, USA^45^ and Canada^32^. The reason for the association will be the nature of the disease where DM is primarily a genetic disease.

Our finding suggests that the magnitude of undiagnosed DM in Ethiopia needs the attention of public health agencies and efforts should be directed to design a feasible means of establishing a system that enables community screening specifically targeted on those with family history of DM, which is useful for early diagnosis and prevention of premature death from complications due to undiagnosed DM. This study is mostly useful for health care workers and planners who want to invest time and other resources in minimizing community deaths due to undetected diabetes, as well as increasing productivity by maintaining population health and lowering health-care costs. If we are unable to do so, it will have a negative impact on people's quality of life and, as a result, on the country's economic progress.

### Limitation of the study

Sub-group analysis could not identify the source of heterogeneity. Studies published in English language included which might not have traced all studies.

## Conclusion

In this review, the pooled prevalence of undiagnosed DM and impaired fasting glucose was significantly high. Family history of DM was statistically significant factors for diabetes mellitus. Based on the findings of this study, we suggest devising a feasible method for developing a community screening system that is explicitly aimed at those with a family history of diabetes, which is useful for early detection and preventing premature death from complications caused by undiagnosed diabetes.

## Data Availability

All data is available in the manuscript.

## References

[1] Tabák AG, Herder C, Rathmann W, Brunner EJ, Kivimäki M (2012). Prediabetes: A high-risk state for diabetes development. Lancet.

[2] Bonora E, Tuomilehto J (2011). The pros and cons of diagnosing diabetes with A1C. Diabetes Care.

[3] WHO. *Definition and diagnosis of diabetes mellitus and intermediate hyperglycemia*. Int Diabet Fed. (2006).

[4] World Health Organization (2016). Global report on diabetes. Isbn.

[5] Al-Daghri NM, Al-Attas OS, Alokail MS, Alkharfy KM, Yousef M, Sabico SL, Chrousos GP (2011). Diabetes mellitus type 2 and other chronic non-communicable diseases in the central region, Saudi Arabia (Riyadh cohort 2): A decade of an epidemic. BMC Med..

[6] Piette JD, Kerr EA (2006). The impact of comorbid chronic conditions on diabetes care. Diabetes Care.

[7] Tunceli K, Bradley CJ, Nerenz D, Williams LK, Pladevall M, Lafata JE (2005). The impact of diabetes on employment and work productivity. Diabetes Care.

[8] Wolde HF (2020). High hidden burden of diabetes mellitus among adults aged 18 years and above in urban northwest Ethiopia. J. Diabetes Res..

[9] Kirigia JM, Sambo HB, Sambo LG, Barry SP (2009). Economic burden of diabetes mellitus in the WHO African region. BMC Int. Health Hum. Rights..

[10] Abdulai T, Li Y, Zhang H, Tu R, Liu X, Zhang L (2019). Prevalence of impaired fasting glucose, type 2 diabetes and associated risk factors in undiagnosed Chinese rural population: The Henan Rural Cohort Study. BMJ Open.

[11] Özdemir L, Topçu S, Nadir I, Nur N, Arslan S, Sümer H (2005). The prevalence of diabetes and impaired glucose tolerance in Sivas, Central Anatolia, Turkey. Diabetes Care.

[12] Uloko AE, Musa BM, Ramalan MA (2018). Prevalence and risk factors for diabetes mellitus in Nigeria: A systematic review and meta-analysis. Diabetes Ther..

[13] Alemayehu Z, Zekewos A, Loha E, Egeno T, Wubshet K, Merga Z (2018). Prevalence of diabetes mellitus and associated factors in southern Ethiopia: A community based study. Ethiop. J. Health Sci..

[14] Worede A, Alemu S, Gelaw YA, Abebe M (2017). The prevalence of impaired fasting glucose and undiagnosed diabetes mellitus and associated risk factors among adults living in a rural Koladiba town, northwest Ethiopia. BMC Res. Notes..

[15] Zhang Y, Pan X, Chen J, Xia L, Cao A, Zhang Y (2020). Combined lifestyle factors and risk of incident type 2 diabetes and prognosis among individuals with type 2 diabetes: A systematic review and meta-analysis of prospective cohort studies. Diabetologia.

[16] Colberg SR, Sigal RJ, Yardley JE, Riddell MC, Dunstan DW, Dempsey PC (2016). Physical activity/exercise and diabetes: A position statement of the American Diabetes Association. Diabetes Care.

[17] Pulgaron ER, Delamater AM (2015). Obesity and type 2 diabetes in children: Epidemiology and treatment. Curr. Diab. Rep..

[18] Hoy D, Brooks P, Woolf A, Blyth F, March L, Bain C (2012). Assessing risk of bias in prevalence studies: Modification of an existing tool and evidence of interrater agreement. J. Clin. Epidemiol..

[19] DerSimonian R, Laird N (1986). Meta-analysis in clinical trials. Control Clin. Trials..

[20] Huedo-Medina TB, Sánchez-Meca J, Marín-Martínez F, Botella J (2006). Assessing heterogeneity in meta-analysis: Q statistic or I 2 Index?. Psychol. Methods..

[21] Duval S, Tweedie R (2000). A nonparametric, “Trim and Fill” method of accounting for publication bias in meta-analysis. J. Am. Stat. Assoc..

[22] Moher D, Liberati A, Tetzlaff J, Altman DG, Altman D, Antes G (2009). Preferred reporting items for systematic reviews and meta-analyses: The PRISMA statement. PLoS Med..

[23] Abebe SM, Berhane Y, Worku A, Assefa A (2014). Diabetes mellitus in North West Ethiopia: A community based study. BMC Public Health.

[24] Dereje N, Earsido A, Temam L, Abebe A (2020). Prevalence and associated factors of diabetes mellitus in Hosanna Town, Southern Ethiopia. Ann. Glob. Health.

[25] Wondemagegn AT, Bizuayehu HM, Abie DD, Ayalneh GM, Tiruye TY, Tessema MT (2017). Undiagnosed diabetes mellitus and related factors in East Gojjam (NW Ethiopia) in 2016: A community-based study. J. Public Health Res..

[26] Endris T, Worede A, Asmelash D (2019). Prevalence of diabetes mellitus, prediabetes and its associated factors in Dessie Town, Northeast Ethiopia: A community-based study. Diabetes Metab. Syndr. Obes. Targets Ther..

[27] Bantie GM, Wondaye AA, Arike EB, Melaku MT, Ejigu ST, Lule A (2019). Prevalence of undiagnosed diabetes mellitus and associated factors among adult residents of Bahir Dar city, northwest Ethiopia: A community-based cross-sectional study. BMJ Open.

[28] Ayele BH, Roba HS, Beyene AS, Mengesha MM (2020). Prevalent, uncontrolled, and undiagnosed diabetes mellitus among urban adults in Dire Dawa, Eastern Ethiopia: A population-based cross-sectional study. SAGE Open Med..

[29] Tesfaye T, Shikur B, Shimels T, Firdu N (2016). Prevalence and factors associated with diabetes mellitus and impaired fasting glucose level among members of federal police commission residing in Addis Ababa. BMC Endocr. Disord..

[30] Zhang N, Yang X, Zhu X, Zhao B, Huang T, Ji Q (2017). Type 2 diabetes mellitus unawareness, prevalence, trends and risk factors: National Health and Nutrition Examination Survey (NHANES) 1999–2010. J. Int. Med. Res..

[31] Al-Lawati JA, Al Riyami AM, Mohammed AJ, Jousilahti P (2002). Increasing prevalence of diabetes mellitus in Oman. Diabet. Med..

[32] Rosella LC, Lebenbaum M, Fitzpatrick T, Zuk A, Booth GL (2015). Prevalence of prediabetes and undiagnosed diabetes in Canada (2007–2011) according to fasting plasma glucose and HbA1c screening criteria. Diabetes Care.

[33] Asmelash D, Getnet W, Biadgo B, Ambachew S, Melak T, Melese L (2018). Undiagnosed diabetes mellitus and associated factors among psychiatric patients receiving antipsychotic drugs at The University of Gondar Hospital, Northwest Ethiopia. Ethiop. J. Health Sci..

[34] Mirzaei M, Rahmaninan M, Mirzaei M, Nadjarzadeh A, Dehghani Tafti AA (2020). Epidemiology of diabetes mellitus, pre-diabetes, undiagnosed and uncontrolled diabetes in Central Iran: Results from Yazd health study. BMC Public Health.

[35] Bener A, Zirie M, Janahi IM, Al-Hamaq AOAA, Musallam M, Wareham NJ (2009). Prevalence of diagnosed and undiagnosed diabetes mellitus and its risk factors in a population-based study of Qatar. Diabetes Res. Clin. Pract..

[36] Megerssa YC, Gebre MW, Birru SK, Goshu AR, Tesfaye DY (2013). Prevalence of undiagnosed diabetes mellitus and its risk factors in elected institutions at Bishoftu town, East Shoa, Ethiopia. J. Diabetes Metab..

[37] Heidemann C, Du Y, Schubert I, Rathmann W, Scheidt-Nave C (2013). Prävalenz und zeitliche Entwicklung des bekannten Diabetes mellitus: Ergebnisse der Studie zur Gesundheit Erwachsener in Deutschland (DEGS1). Bundesgesundheitsblatt - Gesundheitsforsch - Gesundheitsschutz..

[38] Ministry of Health Malaysia. *Malaysia Health Systems Research Report*. Vol. I (Ministry of Health Malaysia, 2016).

[39] Meme N, Amwayi S, Nganga Z, Buregyeya E (2015). Prevalence of undiagnosed diabetes and pre-diabetes among hypertensive patients attending Kiambu district Hospital, Kenya: A cross-sectional study. Pan Afr. Med. J..

[40] Ahmad J, Masoodi MA, Ashraf M, Rashid R, Ahmad R, Ahmad A, Dawood S (2011). Prevalence of diabetes mellitus and its associated risk factors in age group of 20 years and above in Kashmir, India. Al Ameen J. Med. Sci..

[41] Muyer MT, Muls E, Mapatano MA, Makulo JR, Mvitu M, Kimenyembo W (2012). Diabetes and intermediate hyperglycaemia in Kisantu, DR Congo: A cross-sectional prevalence study. BMJ Open.

[42] Mayega RW, Guwatudde D, Makumbi F, Nakwagala FN, Peterson S, Tomson G (2013). Diabetes and pre-diabetes among persons aged 35 to 60 years in Eastern Uganda: Prevalence and associated factors. PLoS ONE.

[43] Soewondo P, Ferrario A, Tahapary DL (2013). Challenges in diabetes management in Indonesia: A literature review. Glob. Health..

[44] Correr CJ, Coura-Vital W, Frade JCQP, Nascimento RCRM, Nascimento LG, Pinheiro EB (2020). Prevalence of people at risk of developing type 2 diabetes mellitus and the involvement of community pharmacies in a national screening campaign: A pioneer action in Brazil. Diabetol. Metab. Syndr..

[45] Demmer RT, Zuk AM, Rosenbaum M, Desvarieux M (2013). Prevalence of diagnosed and undiagnosed type 2 diabetes mellitus among us adolescents: Results from the continuous NHANES, 1999–2010. Am. J. Epidemiol..

